# The virtual cell based assay: Current status and future perspectives

**DOI:** 10.1016/j.tiv.2017.01.009

**Published:** 2017-12

**Authors:** Rabea Graepel, Lara Lamon, David Asturiol, Elisabet Berggren, Elisabeth Joossens, Alicia Paini, Pilar Prieto, Maurice Whelan, Andrew Worth

**Affiliations:** Chemical Safety and Alternative Methods Unit incorporating EURL ECVAM, Directorate Health, Consumers and Reference Materials, European Commission, Joint Research Centre, Ispra, Italy

**Keywords:** VCBA, Virtual cell, *In vitro*, Nanomaterial, Toxicity, Chemical fate

## Abstract

In order to replace the use of animals in toxicity testing, there is a need to predict *in vivo* toxic doses from concentrations that cause toxicological effects in relevant *in vitro* systems. The Virtual Cell Based Assay (VCBA) estimates time-dependent concentration of a test chemical in the cell and cell culture for a given *in vitro* system. The concentrations in the different compartments of the cell and test system are derived from ordinary differential equations, physicochemical parameters of the test chemical and properties of the cell line. The VCBA has been developed for a range of cell lines including BALB/c 3T3 cells, HepG2, HepaRG, lung A459 cells, and cardiomyocytes. The model can be used to design and refine *in vitro* experiments and extrapolate *in vitro* effective concentrations to *in vivo* doses that can be applied in risk assessment. In this paper, we first discuss potential applications of the VCBA: i) design of *in vitro* High Throughput Screening (HTS) experiments; ii) hazard identification (based on acute systemic toxicity); and iii) risk assessment. Further extension of the VCBA is discussed in the second part, exploring potential application to i) manufactured nanomaterials, ii) additional cell lines and endpoints, and considering iii) other opportunities.

## Highlights

• VCBA as an alternative approach can be applied in the domain of nanotoxicology.
• VCBA can support better testing strategies in acute toxicity.
• Refinement of the VCBA taking into account biological oscillators could improve toxicity prediction.
• Extensions of the VCBA can capture effects related to additional subcellular compartments.

## 1 Introduction

The safe use of chemicals is ensured through the application of regulatory measures based on the identification of their intrinsic hazards or on the probability (risk) that these hazards will manifest themselves under defined conditions of exposure. While the management of chemicals involves policy decisions based on multiple considerations, the underpinning safety assessments are scientifically based. In particular, the risk assessment process consists of four steps: (i) hazard identification, (ii) hazard characterisation, (iii) exposure assessment, and (iv) risk characterisation. To determine the safety of chemicals, risk is evaluated as a function of both the exposure and hazard. Traditionally, hazard identification has been based on the use of animal testing, but these methods are gradually giving way to alternative (non-animal) approaches for scientific, ethical and economic reasons. These approaches, typically *in vitro* methods, *in silico* models, or integrated testing strategies (ITS) comprising both *in vitro* and *in silico* components, are increasingly being used as replacement methods, thereby contributing to the practical implementation of the “Three Rs” (Replacement, Reduction and Refinement of animal studies, Russell and Burch, 1959) in toxicology. These non-animal methods are used not only within the chemical and pharmaceutical sectors to support the identification and development of new chemical entities, but also to support the safety assessment of regulated chemicals, and to identify the need for risk management measures in cases of a sudden incident or crisis (*e.g.* food contamination or chemical spill). Reviews on the current status of alternative methods and their use in different sectors are given elsewhere (European Food Safety Authority, 2014, Prieto et al., 2014, Worth et al., 2014).

In order to replace the use of animals in toxicity testing, there is a need to predict *in vivo* toxic doses from concentrations that cause toxicological effects in relevant *in vitro* systems. The use of *in vitro* effect data (*e.g.* perturbation of a molecular pathway or functional read-out) to predict *in vivo* toxicity presents two challenges: first, in analysing the results of *in vitro* experiments, since “nominal” concentrations do not represent the real concentration experienced by the cell (Adler et al., 2011, Broeders et al., 2013, Kramer et al., 2015); and, second, in extrapolating *in vitro* effects to humans, since the true concentration experienced by cells within the target organ is more relevant for human toxicity assessment (Hamon et al., 2015, Yoon et al., 2015). The use of the nominal concentration introduces an uncertainty since in an *in vitro* experiment the chemicals that are tested not only make contact with the cells but can attach to the plastic well, can evaporate, or remain in the media (binding to protein, lipids and other micronutrients). For example, in the case of caffeine, in the liver cell line HepaRG, the amount of test chemical that is freely available (dissolved) in medium after 24 h is 93% (results not shown). On the other hand Amiodarone shows an 85% and 5% affinity to lipids and plastic, respectively. In general the solubility, lipophilicity and volatility of the compound can influence the overall kinetics of the compound in an *in vitro* system. These uncertainties are chemical dependent and can be reduced by using a model that predicts the concentration of chemical in media, cell, plastic, *etc.* by considering only physicochemical properties of the test chemical and some parameters specific to the given cell line.

**Fig. 1:**
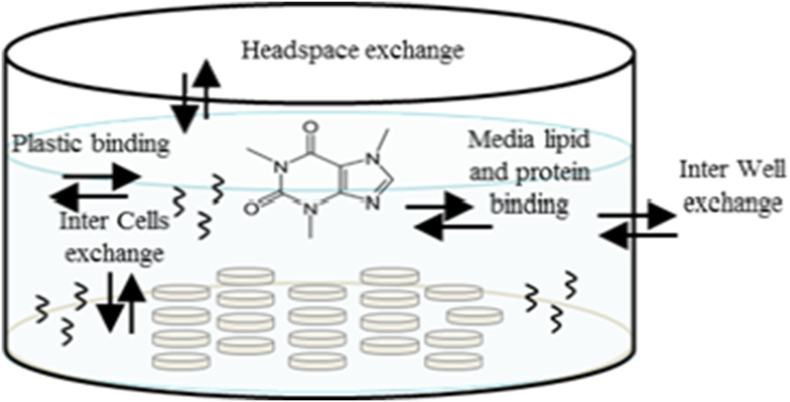
Schematic representation of the virtual cell based assay.

To address the first of these challenges (analysis of *in vitro* experiments) we have developed a Virtual Cell Based Assay (VCBA)^2^ , Fig. 1, which is currently applicable to a range of cell lines (BALB/c 3T3 cells, HepG2, HepaRG, lung A459 cells, cardiomyocytes). The VCBA model consists of ordinary differential equations whose solution allows the calculation over time of the dissolved concentration of a chemical in cell culture as well as the internal concentration in the cells.

**Fig. 2:**
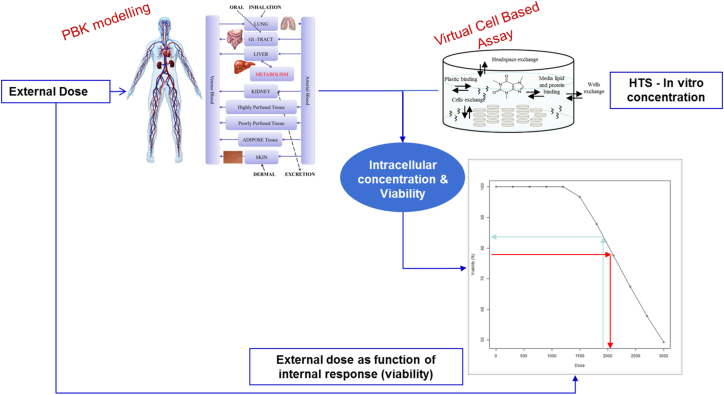
Representation of the process leading from *in vitro* concentrations to *in vivo* doses that are a function of the internal response, such as viability.

To address the second challenge (*in vitro* to *in vivo* extrapolation, IVIVE, Fig. 2) we have developed a series of human PBK models and coupled them with the VCBA (Gajewska et al., 2015). PBK models also consist of a set of differential equations that are typically used to estimate the concentration-time profiles in different tissues/organs within a body based on a known external dose (or exposure pattern), or to estimate the external dose that would result in the effective concentration in the target tissue, based on the known effective concentration determined in a relevant *in vitro* system (Blaauboer, 2008, Blaauboer, 2010, Pelkonen, 2010).

As described in detail in this special issue (Zaldívar et al., 2016), the VCBA model comprises four interconnected models:

1. A fate and transport model that calculates the time-dependent chemical concentration in the medium as well as in the headspace. This takes into consideration a series of processes including evaporation, partitioning of chemicals from the dissolved phase to serum proteins and lipids, adsorption onto the plastic, and also degradation and metabolism.
2. A cell partitioning model that is built on the assumption that once the chemical is taken up by the cell, a partitioning occurs between three compartments: one aqueous fraction and two non-aqueous fractions corresponding to structural components (proteins) and energy resources (lipids).
3. A cell growth and division model that is based on a four stage based approach, with each stage corresponding to one of the four cell cycle phases: G1, S, G2 and M.
4. A toxicity and effects model. The direct effects of a chemical concentration, C, on cell dynamics (survival/mortality) are expressed by using the killing rate, kr, and the no effect concentration, NEC.

In this paper, we discuss the current status of the VCBA and describe three applications that are already technically feasible, at least for selected compounds and cell types: i) the design of *in vitro* High Throughput Screening (HTS) experiments; ii) hazard identification (based on acute systemic toxicity); and iii) risk assessment. We also discuss the scientific and technical developments that will be needed to transform the VCBA from a research tool to one that is accessible to a broad community of safety assessors and decision makers. In this context, we consider the need to extend the applicability of the VCBA to simulate the fate and effects of a wider range of chemicals, including nanomaterials, which pose specific challenges due to the unique properties of substances at the nanoscale.

## 2 Overview of VCBA-like models

The VCBA model consists of ordinary differential equations whose solution allows the estimation over time of the dissolved concentration of a chemical in cell culture as well as the internal concentration in the cells. The VCBA developed by Zaldívar et al. (2016) and referred in this paper is not the only model of its kind. As summarised in Table 1, we have identified at least nine other studies that have developed mathematical models for simulating the fate of chemicals in *in vitro* systems. A few of these studies also include a dynamic endpoint, such as cell viability. As can be seen, the models are based on both mammalian systems (primary cells and cell lines) as well as fish cell lines. Most studies have focused on single dose exposure scenario, with a few exceptions dealing with repeated exposure. Test compounds include pharmaceuticals, industrial chemicals, and pesticides. The Zaldívar et al., 2011, Zaldívar et al., 2012 VCBA was described for use in a single exposure mode for a range of chemicals, in a second stage of the VCBA development we addressed simulation of repeated exposure (Paini et al., 2016). The novelty of the Zaldívar et al. (2016) model is the inclusion in one model of four interconnected sub models describing chemical fate, cell partitioning, cell growth and effect. The previous model so far addressed chemical fate and effect, but not cell partitioning and cell growth.

**Table 1:** VCBA-like models of *in vitro* test systems in the literature.

Model compartments	Cell type	Test Compound	Exposure	Details	Reference
Fate model [five compartments: air, medium, protein, plastic, cell] + cell partitioning model + cell growth model + effect	HepaRG, HepG2 cell line	Amiodarone, caffeine	Single, repeated	Kinetics, dynamics (cell viability)	Paini et al., 2016
Fate model [three compartments: cell, medium, protein] + effect	Primary rat hepatocytes, primary human hepatocytes, HepaRG cell line	Ibuprofen	Single, repeated	Kinetics, dynamics (cell viability)	(Truisi et al., 2015)
Fate model [three compartments: cell, plastic and medium]	Rainbow trout (*Oncorhynchus mykiss*) cell line RTgill-W1	Imidacloprid, Dimethoate, Carbendazim, Malathion, Cyproconazole, Propiconazole, Pentachlorophenol, Cypermethrin, 1,2,3-Trichlorobenzene, Naphtalene, Hexachlorobenzene	Single	Kinetics	(Stadnicka-Michalak et al., 2014)
Fate model [two compartments: medium, cell] + sub compartment metabolic clearance	Primary rat hepatocytes, Primary human hepatocytes, HepaRG cell line	Chlorpromazine	Single, repeated	Kinetics	(Broeders et al., 2014)
Fate model [three compartments: head space, medium + sub compartment serum, plastic, DMSO, dissolved organic matter, and cell/tissue]	Any	Any	Single	Kinetics	(Armitage et al., 2014)
Fate model [five compartments: air, medium, protein, plastic, cell] + cell partitioning model + cell growth model + effect	Lung cell line A549	HPTE 2,2-Bis(4-hydroxyphenyl)-1,1,1-trichloroethane, Pyraclostrobin, Diquat dibromide, Abamectin, Bisphenol, Benomyl	Single	Kinetics, dynamics	(Zaldívar et al., 2012)
Fate model [five compartments: air, medium, protein, plastic, cell] + effect	BALB/c 3T3 cell line RTgill-W1 cell line	Phenanthrene	Single	Kinetics, dynamics (cell viability	(Kramer et al., 2012)
Fate model [five compartments: air, medium, protein, plastic, cell] + cell partitioning model + cell growth model + effect	BALB/c 3T3 cell line	Acetaminophen, Verapamil hydrochloride, Acetylsalicylic acid, Maprotiline, Cycloheximide, Sodium lauryl sulfate, tert-Butyl hydroperoxide, Valproic acid, Rifampin, Thioridazine hydrochloride, Amiodarone hydrochloride, Caffeine, Carbamazepine, Colchicine, Acrylamide, Diphenhydramine, Pentachlorophenol, Disopyramide, Chloroquine diphosphate, Tetracycline hydrochloride.	Single	Kinetics, dynamics	(Zaldívar et al., 2011)
Fate model [five compartments: air, medium, protein, plastic, cell] + cell model + cell growth model	BALB/c 3T3 cell line	Polycyclic Aromatic Hydrocarbons (PAH)	Single	Kinetics	(Zaldívar et al., 2010)
Fate model [five compartments: air, medium, protein, plastic, cell] + effect	Rainbow trout (*Oncorhynchus mykiss*) cell lines RTL-W1 and RTgill-W1	Benzo(a)pyrene, 1,2-dichlorobenzene, 1,2,4-trichlorobenzene	Repeated	Kinetics, dynamics (cell viability)	(Kramer et al., 2010)
Fate model [two compartments: plasma and response tissue]	MCF-7 cells	Genistein, bisphenol A, and octylphenol			(Teeguarden and Barton, 2004)
Fate model [two compartments: medium, cell]	Human embryonal kidney cell line (HEK293)	[3*H*]estradiol, octylphenol.	Single	Kinetics	(Heringa, 2004)
Fate model [three compartments: Cells, culture medium and culture vessel]	Sperm cells	Antimycin A, digitonin, thioridazine HCl, hexachlorophene 4,4′-DDE, dieldrin, pentachlorophenol, methylmercury chloride, xylene, 1-nitronaphthalene	Single	Kinetics	(Gülden et al., 2001)

## 3 Potential applications of the VCBA model

### 3.1 Design of *in vitro* (HTS) experiments

High-throughput screening (HTS) has proven to be a useful technique used in drug design and may be applied in biological and chemical sciences (Szymański et al., 2011). Using robotics, data processing and control software, liquid handling devices and sensitive detectors, HTS allows to quickly conduct a number of biochemical, genetic or pharmacological tests. Through this process one can rapidly identify active compounds, antibodies or genes which modulate a particular biomolecular pathway. The results of these experiments provide starting points for drug design and for understanding the biological role of a particular biochemical process. This analytical technique can also be applied in the hazard identification and (dose-response) characterisation of toxic substances. The use of HTS in combination with High Content Imaging (HCI) to screen environmental chemicals for mechanistic (toxicokinetic and toxicodynamic) properties underlying adverse effects is being used to support regulatory decision making on chemicals (Kavlock et al., 2012, Tice et al., 2013).

The current VCBA can support HTS by:

1) Optimizing the efficiency of HT chemical-design methods (Seifert et al., 2003); for instance, testing several experimental settings within the VCBA first would give inside on (several) expected outcome/s. Based on the results obtained the HTS experimental design can then be optimized in such a way that the desired effect can be best observed.
2) Extracting additional information from the HTS process. When screening multiple compounds applying HTS, this allows only one experimental design and does not allow modifications on a compound by compound basis which otherwise would be included to capture specific behaviour. However, this extraction can be obtained after the HTS is done, as the data of the HTS experiment allows the full parameterisation of the VCBA model for each compound which can then be used to model the compound specific exposure.
3) Helping the interpretation of unexpected False Negative effects within the experiment as this could be due to the *in vitro* kinetics.

### 3.2 Hazard identification and classification - acute systemic toxicity

The VCBA model has already been developed and implemented for several cell lines (such as HepaRG, 3T3 Balb/c, cardiomyocytes, A539) and 32 chemicals including polyaromatic hydrocarbons, cosmetics, and drugs (caffeine, anthracene, amiodarone, sodium lauryl sulfate, etc.) that span molecular weight from 71 to 822 g/mol and octanol-water partition coefficient from − 3.7 to 7.6. The VCBA simulates cell viability, in addition to the distribution of the chemical in an *in vitro* experimental setup (media, plastic, intracellular concentration). Simulations of cell viability/cytotoxicity can be performed if the toxicodynamic part of the VCBA has been calibrated with *in vitro* experimental data. Information on the kinetics in an *in vitro* test system are highly relevant to refinement of any additional *in vitro* tests, and their application could help to better understanding and translating information to address acute oral toxicity (Bhhatarai et al., 2015).

Acute systemic oral toxicity represents a complex *in vivo* endpoint routinely required for hazard identification and classification purposes under multiple pieces of EU legislation (CLP, REACH, Pesticides, Biocides, and Cosmetics; Prieto et al., 2014). Yet there is no officially accepted non-animal alternative approach, despite decades of research and development. Acute systemic toxicity testing is carried out to identify the dose or concentration at which a single exposure to a test compound *via* the oral, dermal or inhalational route, results in severe adverse effects (death) in the test population within a 14 day observation period (Prieto et al., 2014). The outcome of the assay, the LD_50_/LC_50_ value, is then used to determine the toxic class of the compound in order to identify hazards and manage risks (EU Regulation on Classification, Labelling and Packaging).

One of the challenges in this field is to predict acute toxic classes using alternative methods at least as accurately as the current *in vivo* tests do. The use of basal cytotoxicity to predict acute oral toxicity *in vivo* has been extensively studied (Combes et al., 2008). The ACuteTox project (http://www.acutetox.eu/) evaluated the use of a battery of *in vitro* and *in silico* tests to predict acute oral systemic toxicity. While even the use of multiple, combined *in vitro* methods did not allow the correct identification of the different toxic classes according to CLP, some of the organ specific methods, such as the brain aggregate model, could be used to alert for specific target organ toxicities (Prieto et al., 2013, Zurich et al., 2013). However, the results from the basal cytotoxicity assay, *i.e.* the BALB/c 3T3 neutral red uptake (NRU) assay, are useful in identifying non-classified (LD_50_ > 2000 mg/kg) substances according to CLP. Oral LD_50_ values can be derived from IC_50_ values that are produced in the *in vitro* 3T3 NRU experiments (Interagency Coordinating Committee on the Validation of Alt, Prieto et al., 2013). When interpreting these results, it is important to keep in mind some of the limitations of *in vitro* studies such as the lack of metabolic competence of the 3T3 cell line as well as considerations of *in vitro* kinetics. In addition, the variability of the reference data needs to be taken into consideration (Hoffmann et al., 2010).

The VCBA has been implemented for the 3T3 cell line and the neutral red uptake (NRU) assay. Simulations have been made for 32 compounds, after single exposure, for which available experimental data to perform downstream extrapolation were available (Zaldívar et al., 2010, Zaldívar et al., 2011). For each of the 32 compounds, physicochemical properties were collected from EPIsuite or Chemspider. The two parameters describing the toxicity (killing rate and the no effect concentration) were optimized using *in vitro* 3T3 NRU data by applying the minimal square error (for more information on the model applicability and how to perform simulation and optimization please refer to Zaldívar et al., 2016).

The VCBA simulates cell viability in addition to calculating the theoretical, corresponding intracellular concentration and the distribution of the test chemical in the *in vitro* set up. Knowing the time-dependent concentration of chemical at different levels of the *in vitro* test system can be of great help in detecting false negative predictions as there are cases in which a negative result is due to the chemical not reaching the target at a sufficient concentration due to its fate in the system (*e.g.* binding to plastic, evaporation to the overhead space). In this regard, and in the context of the published EURL ECVAM strategy on how to reduce, refine and ultimately avoid the use of animals for acute mammalian systemic toxicity testing (Prieto et al., 2014), we are currently investigating the application of the VCBA model to increase the confidence in the *in vitro* negative results (*e.g.* predicted oral LD_50_ > 2000 mg/kg, see Prieto et al., 2013, Prieto et al., 2014) using a larger set of compounds for which 3 T3 NRU cytotoxicity values have been obtained experimentally. An easy screening system consists in comparing the simulated and the nominal concentrations of the dissolved chemical, a compound for which the *in vitro* kinetics would have a clear influence on the cytotoxicity result could be flagged. Efforts are still needed to prove that the VCBA simulations are relevant to predict acute oral toxicity for different regulatory contexts. A recent report by a US Committee on Predictive Toxicology approaches for Military Assessments of Acute Exposures, discussed the challenges of integrating information from different alternative methods such as QSAR predictions and *in vitro* tests for basal cytotoxicity and cell specific mechanisms in order to form one overall prediction of toxicity (National Academies of Sciences Engineering and Medicine, 2015).

### 3.3 Integration of the VCBA into a safety assessment framework

For prediction and extrapolation of *in vitro* concentrations to *in vivo* doses, it will be necessary to couple the VCBA with a human Physiologically Based Kinetic (PBK) model. This may contribute to the chemical risk assessment process. As outlined above, many kinetic considerations have to be taken into account when extrapolating from an *in vitro* nominal concentration to an *in vivo* concentration. Thus, the kinetics of an *in vitro* test system has to be considered, as well as the kinetics of an *in vivo* exposure, when trying to translate an *in vitro* dose to an *in vivo* dose.

The starting point in a completely animal-free risk assessment is to analyse expected exposure scenarios and predict the relevant dose and route of exposure. Then to better understand possible effects and target organs, it is necessary to collect current knowledge and search for already existing animal and *in vitro* data, for the substance to be assessed or structurally similar substances. For example, the publicly available ToxCAST library (US EPA, 2014) is including data from a large number of *in vitro* assays and chemicals and can provide useful information for setting up a hypothesis on possible target tissues, as well as historical animal data. Further physicochemical data and chemometric analysis will provide knowledge to predict the chemical's fate in the human body together with PBK modelling. QSARs can be used to predict possible molecular initial events or reactivity of chemicals. At this stage, it might be possible to predict whether the toxicity is rather general and the effect in different organ cells would basically be the same, or whether a specific organ effect is expected. It might also be possible to understand whether it could be sufficient to apply a Threshold of Toxicological Concern (TTC), or whether further assessment is necessary, in which case it should also carefully be considered whether it would be possible to use any read-across assessment.

Depending on the hypothesis regarding target tissues, the VCBA could be adapted to predict expected adverse effects. The VCBA would then assist in predicting any expected adverse effect either a stand-alone assessment, or possibly to strengthen a read-across case.

When trying to extrapolate *in vitro* results to *in vivo* (human) effects, whole-body toxicokinetics (absorption, distribution metabolism and excretion - ADME) needs to be considered. Depending on the route of administration, oral, dermal or inhalation, the chemical first has to cross the relevant barrier to be absorbed into the blood stream if it is to have a systemic effect. This information can be provided by available QSAR models on absorption potential (Butina et al., 2002, Boobis et al., 2002).

The application of biokinetic/biodynamic models is an important means of extrapolating *in vitro* dose – metrics to the *in vivo* situation (Blaauboer et al., 2012). As such, biokinetic models are used to translate exposure metrics, and align responses or biodynamics across the systems. PBK models and the VCBA can be linked to form a so called Physiologically Based Dynamic (PBD) model, in order to simulate a dynamic effect *in vivo*, such as an adverse outcome. This joint (multiscale) modelling approach can be used to perform IVIVE (Teng et al., 2015). Additionally, such an approach was carried out previously by Gajewska et al. (2015) where comparison of the internal concentration between PBK model and VCBA for caffeine was performed in relation to HepaRG cell viability. These models can simulate a full time profile or concentration response curve which can be used to derive a point of departure for risk assessment. For instance, a dose–response curve can be generated by linking the PBK model output (*e.g.* liver C_max_) with the VCBA model, which allows the application of both forward and reverse dosimetry. The forward approach means that when exposed to a given external dose, the joint model predicts the corresponding effect on liver cell viability. The backward approach can be used to estimate from a given cell viability the corresponding external dose. In addition, a user-friendly tool could be developed by implementing the PBK/D model into a KNIME workflow as currently done for the VCBA (Paini et al., 2016). This tool (workflow) is freely and readily available for end-users and can be used to perform IVIVE in support of chemical risk assessment (https://knimewebportal.cosmostox.eu).

When going through the different steps of the assessment, it is a necessary to accompany each step with an analysis of the uncertainty, to estimate the reliability in the final predicted point of departure.

## 4 Technical adaptation of the VCBA model

While the previous section addressed applications that are already feasible with the current version of the VCBA, this section explores possibilities for future versions of the model.

### 4.1 Extension to particles and manufactured nanomaterials

Manufactured nanomaterials (MNs) are materials with one or more dimensions in the nanoscale, (typically in the range 1–100 nm; ISO, 2011). These materials are increasingly being included in a wide variety of products (*e.g.* flame retardants, cosmetics, food packaging, paints), since materials at the nanoscale exhibit novel physical and chemical properties which may enhance the effectiveness of the embedding products (European Commission, 2012). There are concerns, however, that the very same characteristics may also lead to environmental and human health risks. Given the rapidly growing number of MNs entering the market, there is a pressing need to establish scientifically robust and efficient safety assessment approaches. Traditional experimental approaches, and in particular animal studies, are not a viable solution, given the costs, time and ethical concerns involved. For this reason, there has been considerable research over the last decade to develop alternative (non-animal) approaches. These include *in vitro* methods, which contribute to our knowledge of particle-induced cellular responses and modes of action (Maher et al., 2014), computational approaches such structure-property relationships, which capture the relationships between the intrinsic and extrinsic properties of MNs (Lynch et al., 2014), and mechanistically-based mathematical models which capture knowledge on fate and effects (Mukherjee et al., 2014).

In this context, the integration of the VCBA model with PBK modelling would provide a multi-scale model of the fate and effects of MNs in the human body, thereby enabling the IVIVE of (adverse) cellular effects. As reported below, size distribution and surface characteristics of MNs play an important role in the fate of MNs in *in vitro* systems. Experimental methods have been developed and applied for the measurement of agglomerates diameter and density (DeLoid et al., 2014, Cohen et al., 2014), and such databases have supported the validation of *in vitro* dosimetry models for MNs (Teeguarden et al., 2014). Other experimental studies focused on the *in vitro* fate of MNs (Rischitor et al., 2016): thus, it is realistic to consider the extension of VCBA to the field of MNs.

However, for the VCBA to be applicable to MNs, additional fate processes need to be accounted for in the mathematical model, which also implies a need for targeted *in vitro* studies to generate relevant input parameters. The MN-specific fate processes are briefly explained below.

The fate of MNs in *in vitro* systems is affected mainly by the following fate processes: diffusion, gravitational settling, agglomeration, dissolution, and cellular uptake. These are affected not only by the characteristics of particles (*e.g.* size, shape, reactivity, agglomeration state), but also for those of the surrounding medium (chemical and biochemical composition, density, viscosity). These particle-medium interactions, therefore, influence the fate and intracellular (biologically effective) concentration of MNs.

The current version of the VCBA (Zaldívar et al., 2016) takes into account the binding of substances to organic macromolecules (lipids, proteins) and diffusion kinetics, which are also taken into account in cell fate models applied to MNs (Chithrani et al., 2006, Beddoes et al., 2015). Also the processes of transformation (degradation), dissolution, passive diffusion, and cellular uptake and elimination are considered in the VCBA and are applied in models addressing MNs (Teeguarden et al., 2007, Hinderliter et al., 2010, Treuel et al., 2013, Schultz et al., 2015). However, the description of the processes governing MN fate is also dependent on specific properties like shape, size, surface chemistry, fractal dimension, agglomeration, porosity of agglomerates, surface area, all of which would need to be implemented into the VCBA.

Furthermore, considering the models specifically applied to MNs the following processes would need to be implemented in the VCBA:

1) Passive diffusion in fluids is calculated in the VCBA as a function of physicochemical properties of chemicals (molecular weight and dissociation constant, molar volume) and of properties of the fluid (density, viscosity); for particles including MNs size plays a role and passive diffusion can be calculated based on the Einstein-Stokes equation (*e.g.* (Hinderliter et al., 2010).
2) Agglomeration and gravitational settling are relevant processes in predicting MNs fate (Schultz et al., 2015) and are considered in *in vitro* fate models applied to MNs (Teeguarden et al., 2007, Hinderliter et al., 2010, Fröhlich and Salar-Behzadi, 2014); these processes are not included in the VCBA and, thus, would need to be implemented when applying the model to the case of MNs.
3) There is evidence that size, surface charge, shape (Oh and Park, 2014), surface chemistry (Chithrani et al., 2006), zeta potential (Teeguarden et al., 2007) and protein binding (Schultz et al., 2015) affect cellular uptake of MNs; size, surface charge shape surface chemistry and zeta potential would also need to be included in the VCBA for application to the MNs case study.

### 4.2 Extension to additional cell lines and endpoints

In its current form the VCBA has been implemented for single (and sometimes repeated) exposures in a limited number of cell lines. In order to use the VCBA to support the replacement of *in vivo* systemic toxicity tests it would need to be extended to a broader range of cell lines and endpoints. This is discussed here in relation to acute systemic toxicity, as an endpoint that likely involves more than one target organ. It is worth noting that while this discussion is focused on the identification of other cell types to be implemented in the VCBA; there are of course other variable experimental parameters such as new medium composition and different types of plastic that may be considered in future versions of the VCBA.

It has been suggested that acute systemic toxicity could be due to organ specific effects in addition to basal cytotoxic effects (Gennari et al., 2004). Thus implementing the VCBA for those target organs might improve prediction of acute systemic toxicity of a new chemical. In order to do this, we need to identify cell lines that are representative of potential target organs and also discuss whether endpoints such us cytotoxicity are still suitable. First of all, the potential target organs where damage could lead to acute systemic toxicity were identified by Gennari et al. (2004) as: the blood, the lungs, the liver, the gastrointestinal tract, the nervous system, the cardiovascular system, the kidneys and the immune system. In order to implement the VCBA for a target organ it is necessary to identify the cell types that are present in it, determine the most representative and toxicologically relevant cell types and then gather *in vitro* data for these cell lines in order to train the VCBA model. Table 2 provides an overview of the potential target organs, their principal cell types and information on whether a cell type has already been implemented for the VCBA.

**Table 2:** Overview of potential acute systemic toxicity target organs.

Target organ	Main cell types	VCBA already implemented?	Cell lines available (commercially)?
Blood	• Erythrocytes • Leukocytes • Thrombocytes	No	Human or animal whole blood
Lungs	• Alveolar type I & II endothelial cells • Capillary endothelial cells • Cells in the interstitial space • Alveolar macrophages	Yes - A549 (adenocarcinomic human alveolar basal epithelial cells)	Yes
Liver	• Hepatocytes • Endothelial cells • Kuppfer cells • Stellate cells	Yes – HepG2 & HepaRG	Yes
Gastrointestinal tract	• Epithelial cells • Paneth cells • Enterocytes • Goblet cells • Enteroendocrine cells • Stem cells	No	Yes
Nervous system	• Neurons • Microglia • Astrocytes • PNS: Oligodendrocytes • Schwann cells • Satellite cells	No	Yes
Cardiovascular system	• Heart: Cardiomyocytes • Sino atrial and atrioventricular nodes • Purkinji fibers • Vaculature: Vascular smooth muscle cells • Endothelial cells • Blood Brain Barrier: Pericytes • Fibroblasts • Astrocytes	Yes - cardiomyocytes	Yes
Kidneys	• Epithelial cells (glomerular, squamous or cuboidal) • Capillary endothelial cells • Juxtaglomerular cells • Podocytes • Macula densa cells • Extraglomerular mesangial cells • Brushborder cells • Intercalated cells (α, β) • Connecting tubule cells	No	Yes
Immune system	• Neutrophils • Eosinophils • Basophils • Mast cells • Dendritic cells • Macrophages • Natural killer cells • B cells • T cells • Tissue specific resident immune cells	No	Yes

Table 2 demonstrates some of the complexity faced by researchers who are striving to find *in vitro* test methods for systemic toxic endpoints. Each organ is comprised of several cell types that work together to make the organ function. In terms of modelling the function of the organ, is it then advisable to model only the cell type that makes up the largest portion of the organ? For example, the liver is comprised to 60% of hepatocytes when determined by absolute cell number, but make up 80% of the organ by volume. In some cases that might be the ideal cell type to model since its function might determine the overall function of the organ. However, for other organs/systems, such as the immune system it is not so clear which cell type would be most representative/most vulnerable to toxic insult/ or which feature should be modelled. For most of these organs, human and animal cells for at least one cell type, either primary or immortalised, are available commercially to be cultured and tested in the laboratory. For blood cells it is possible to obtain whole human blood from donors. In addition, advances in stem cell research have made the *in vitro* production of red blood cells from stem cells a possibility (Migliaccio et al., 2012). The availability of standardised cell culture systems is an important consideration in extending the VCBA model to more cell types, since high quality *in vitro* data needs to be generated in order to train the model.

It is also important to consider the endpoint(s) that should be investigated. While cytotoxicity still plays a role in target organ toxicity, it is also necessary to consider whether target organ specific mechanisms of toxicity play an additional (and possibly more important) role. For example, the neurons of the central and peripheral nervous system communicate through the generation and propagation of action potentials and the release of neurotransmitters. Toxicants can interfere with this basic function of the cell at several levels: the generation and propagation of the action potential is governed by the opening and closing of ion channels, with the ion balance across the membrane being restored by transporter enzymes; neurotransmitters are produced in the neuron and released from the nerve ending where they interact with their target receptors before they are cleared from the synaptic cleft. Interference with any of these steps would inhibit the normal functioning of the nervous system and thus could lead to death or severe adverse effects. Ongoing work is focused on trying to determine the mechanisms that lead to acute systemic toxicity. For example, information gained from *in vitro* test systems such as the multielectrode array (MEA) chips, which record electrical activity from cultured cortical neurons in the presence of test substances, could provide important functional information in this context (Scelfo et al., 2012). In terms of the VCBA, it will be very challenging to implement it for some test systems. As outlined above there are numerous cell types with a great number of outcomes that may need to be modelled, for instance representing the shape of a neuron mathematically is not an easy task. Nonetheless, a simplified version of the VCBA for neurons, taking an average shape of neurons from a population, could be developed to start taking into account neurotoxic effects.

### 4.3 Other opportunities for conceptual and technical development of the VCBA

Other opportunities for further developing VCBA, both in terms of its mathematical formulation and technical implementation, include: 1) full automation of the model; 2) extensions of the fate (kinetic) models; 3) modifications of the toxicity (dynamic) model, and 4) embedding the cellular component of the VCBA, *i.e.* the virtual cell, in larger scale models of organs and the human body; 5) incorporating biological oscillators; and 6) accounting for the influence of new medium composition and plastic/coating.

1) In its current state, the model that is available as a KNIME workflow requires the user to provide a series of parameters for each of the chemicals that need to be tested. This does not preclude the use of the model, but represents an inconvenience for users that do not dispose of the necessary experimental values and need to obtain them elsewhere, *e.g. via* Quantitative Structure-Property Relationships (QSPR). In the worst case scenario, two different users that provide parameters for the same chemical obtained from different QSPRs could obtain different results. In order to tackle this issue, a node that will calculate the necessary parameters from just the name, CAS number, or SMILES codes of the test chemical will be added to the current workflow. The node will also allow the user to provide its own parameters if desired. The definition of model applicability domain (AD) is key for the use of QSARs and QSPRs as it determines whether the QSAR/QSPR is appropriate for the test chemical, and whether there is confidence in the resulting prediction. The fact that a physicochemical property is obtained from a QSPR for which the test chemical falls outside the AD does not necessarily imply that the value is wrong, but it means that the model was not optimized for such chemicals and that the quality of the prediction is uncertain. In order to gain confidence in the physicochemical predictions and the overall VCBA result simulation, the new implementation will also provide an alert indicating the test chemicals that fall outside the AD of the corresponding QSPR so that the user is aware of the increase of uncertainty of the result. Such an automation of the calculations will benefit the model in two ways: a) it will allow the model to be used in a batch mode, b) will reduce the uncertainties caused by the need to use third party software to obtain parameters. Thus, such a node will contribute to a better user experience and to increase the traceability of the model. The parameters needed to run the VCBA are the molar volume at its normal boiling point (Vb), Henry law constant (H), octanol-water partition coefficient (K_ow_), and the air and water degradation constants (K_AW_). The parts of the model that make use of these parameters can be found in another article that forms part of this special issue (Zaldívar et al., 2016).
2) Possible extensions of the fate models were discussed above in relation to nanomaterials, the fate of which in *in vitro* test systems and cells depends on additional physical processes. More generally, additional subcellular compartments could also be considered, for example, just as a generic mitochondrial compartment has been implemented, a generic lysosomal compartment could be added. This could be useful to capture the direct and indirect effects of chemicals, including MNs, on the lysosomes. Similarly, consideration could be given to adding compartments for the nucleus to simulate genotoxic effects, and the endoplasmic reticulum, to simulate for example effects on protein folding (Griesemer et al., 2014, Wang and Kaufman, 2014).
3) Possible extensions of the toxicity and effects model could be to capture effects (*in vitro* readouts) related to additional subcellular compartments. Another suggestion relates to the use of NEC and kr as the effect parameters. In the current version of the VCBA, the dose-response of a chemical is expressed as an exponential function of the exposure concentration (Zaldívar et al., 2016). This assumes that all cells in a culture respond equally and proportionately to the concentration. In reality, some cells are more sensitive than others (*i.e.* are “early” responders) and the differences in sensitivity follow a log-normal distribution. Thus, a possible extension would be to incorporate a probabilistic term to capture this natural variability in the susceptibility of cells to a given concentration.
4) Another possible extension of the VCBA is the integration of virtual cells into virtual organs and organisms, so called multiscale modelling. The need to develop a virtual physiological human for different applications has stimulated the development of several physiological models (Diaz Ochoa et al., 2013). These models capture the interplay between different structures in tissues, organs, and the whole body (Fenner et al., 2008). For the liver, prominent examples are the virtual liver project (Shah and Wambaugh, 2010) and the virtual liver network (Holzhütter et al., 2012). In a number of liver modelling efforts, relatively simple cells were coupled to models of liver tissue to perform qualitative predictions of substance distributions and cell responses, particularly toxicity. In some pharmacokinetic models, data obtained from *in vitro* experiments are translated into different transporter and enzyme activities, which are thereafter distributed in the liver (Pang et al., 2007). Other approaches defined complete *in silico* livers where the organ is coupled with a simple model of cell metabolism. Multi-scale models that can be applied for predicting adverse outcome and toxicological risk can consist of detailed mechanistic cellular models integrated into organ models which are embedded in a whole body model (Diaz Ochoa et al., 2013).
5) Oscillations in biological processes are periodic fluctuations ranging from hormonal oscillations with periods of days and months, to genetic fluctuations in the range of hours (*e.g.* circadian clock, embryonic oscillators), and metabolic and biochemical oscillations in the order of minutes (glycolytic oscillations) or seconds (calcium-dynamics). Oscillations are central to biology across different temporal and spatial scales and Zaldívar (2010) made a first attempt to describe how toxic effects propagate from molecular to population level, with a simple model of a circadian oscillator coupled with a cyanobacteria growth model. The novelty of such application consisted in building a hypothesis where the influence of the circadian clock is taken into account in modelling the chemical fate and its effect on the cells system. Biological oscillators are interesting mechanisms that could reveal more information on interactions of chemicals with the human body at different biological scales; this type of mathematical description could have a potential in the development of the VCBA and PBK models.
6) Most of the current well plates are produced using polystyrene. However, since polystyrene has some limitations in its application to the *in vitro* system, the plates are increasingly being coated. Basic synthetic polymers, such as poly-d-lysine (PDL), have also been used as coatings to create a positive charge on polystyrene which, for some cell types, can enhance cell attachment, growth and differentiation, especially in serum-free and low serum conditions (Ryan, 2008). This experimental aspect should also be explored further, to determine whether the experimental set-up component of the VCBA should also be adapted.

## 5 Concluding remarks

Modern toxicity testing is moving away from its traditional reliance on the observation of apical effects in animal studies towards mechanism-based testing strategies that utilise techniques such as *in vitro* HTS, adverse outcome pathways (AOPs) and a range of modern computational tools including the VCBA. The literature includes many variants of this emerging safety assessment framework (SEURAT-1, 2011, Nel et al., 2013, US EPA, 2014, Daston et al., 2015). In the view of the authors, the future of chemical safety assessment involves the phasing out of traditional animal testing based on the principle that an equivalent level of protection of human health and the environment can be achieved more effectively and efficiently through the use of alternative, and in particular non-animal, methods. This does not mean that any individual non-animal method needs to be directly predictive of a given toxicity endpoint in animals, but that the information provided by one or more such methods, for example in the context of an integrated testing strategy, is protective, according to a defined set of protection goals. In other words, we envision a future framework where a set of protection goals is linked to a set of information requirements, which can be fulfilled by using a battery of alternative methods. Within this battery of alternative methods, *in vitro* tests will provide information on key events underlying the relevant AOPs. In addition, computational models will play a crucial role, not only by providing individual pieces of information (*e.g.* QSARs for molecular initiating events) but also by integrating experimental and computable properties in the form of mathematically based phenomenological models. PBK and VCBA models are thus central to this paradigm shift, which is why progress in the scientific and technical development of these approaches needs to be continued, along with efforts directed at promoting their uptake and acceptance among scientist and regulators.

The VCBA, and VCBA-like models, are already being used as research tools to support the interpretation of *in vitro* toxicity data, as well as the design (dosimetry) of *in vitro* experiments. As far as the authors are aware, these models have not yet been used to directly support a regulatory application, such as hazard characterisation, IVIVE, or risk assessment. This paper has identified some of the scientific and technical opportunities and challenges associated with possible applications of the VCBA in regulatory chemical risk assessment. While some of these challenges will require considerable further *in vitro* experimentation, it can be seen from the papers in this special issue that considerable progress has been made in the past five years, not only in developing the computational models and tools, but also in making them available and deployable in a user-friendly form.

## Transparency Document

Transparency document. Image 1

## Footnotes

2 In the Virtual Cell Based Assay (VCBA), the word *virtual* means that it is a theoretical and mathematical model.
